# Investigating discrimination in the workplace. Translation and validation of the Everyday Discrimination Scale for nursing staff in Germany

**DOI:** 10.1186/s12912-023-01367-w

**Published:** 2023-06-09

**Authors:** Nazan Ulusoy, Albert Nienhaus, Patrick Brzoska

**Affiliations:** 1 Institute for Health Services Research in Dermatology and Nursing (CVcare), University Medical Center Hamburg-Eppendorf (UKE), Martinistrasse 52, 20246 Hamburg, Germany; 2 Department of Occupational Medicine, Hazardous Substances and Public Health, German Social Accident Insurance Institution for Health and Welfare Services, 22089 Hamburg, Germany; 3 Health Services Research, Faculty of Health, School of Medicine, Witten/Herdecke University, Alfred-Herrhausen-Straße 50, 58448 Witten, Germany

**Keywords:** Everyday discrimination scale, Validation, German, MIMIC, DIF, Nurses

## Abstract

**Background:**

The Everyday Discrimination Scale (EDS) is a frequently used questionnaire in the field of health and social psychology that aims to explore perceptions of discrimination, especially instances of injustice related to various diversity characteristics. No adaptation to health care staff exists. The present study translates and adapts the EDS to nursing staff in Germany and examines its reliability and factorial validity as well as its measurement equivalence between men and women and different age groups.

**Methods:**

The study was based on an online survey conducted among health care staff of two hospitals and two inpatient care facilities in Germany. The EDS was translated using a forward-backward translation approach. Direct maximum likelihood confirmatory factor analysis (CFA) was conducted to examine the factorial validity of the adapted EDS. Differential item functioning (DIF) related to age and sex was investigated by means of multiple indicators, multiple causes (MIMIC) models.

**Results:**

Data on 302 individuals was available, of whom 237 (78.5%) were women. The most commonly employed one-factor, 8-item baseline model of the adapted EDS showed a poor fit (RMSEA = 0.149; CFI = 0.812; TLI = 0.737; SRMR = 0.072). The model fit improved considerably after including three error covariances between items 1 and 2, items 4 and 5, and items 7 and 8 (RMSEA = 0.066; CFI = 0.969; TLI = 0.949; SRMR = 0.036). Item 4 showed DIF related to sex and age, item 6 showed DIF related to age. DIF was moderate in size and did not bias the comparison between men and women or between younger and older employees.

**Conclusions:**

The EDS can be considered a valid instrument for the assessment of discrimination experiences among nursing staff. Given that the questionnaire, similar to other EDS adaptations, may be prone to DIF and also considering that some error covariances need to be parameterized, latent variable modelling should be used for the analysis of the questionnaire.

## Introduction

Discrimination can occur in all areas of life and may also be encountered in the workplace [1–3]. It can be associated with perceptible characteristics such as age, sex, disability, ethnicity or characteristics which are not immediately perceptible such as religion and belief or sexual identity. Discrimination may manifest in exclusion, insults, sexual harassment or other forms of physical or psychological violence. In Germany, for example, a study conducted by the Federal Anti-Discrimination Agency in 2016 found that almost one-third of all employees in Germany had experienced discrimination during a two-year period. Discrimination based on age (49%) was most prevalent, followed by discrimination based on gender (37%), religion and belief (25%) and ethnicity (21%) [4].

Discrimination in the workplace not only has a direct negative impact on those affected, but also an indirect impact on the entire work environment and corporate culture. It can lead to reduced commitment and motivation of affected employees and to an erosion of trust and cooperation within the company. Discrimination can also contribute to companies becoming less attractive to certain segments of the population, making it difficult to attract and retain qualified employees [5–7].

Although discrimination is also a common problem among health care workers, it has received comparably little attention in research. Existing studies focusing on health care report primarily unequal treatment and discrimination directed at patients [8–10]. However, discrimination can also originate from patients or colleagues and be directed towards health professionals [11–13]. In nursing, discrimination has been studied mainly in the context of minorities and internationally and foreign-trained nurses [14] and in relation to institutional/structural discrimination (lack of opportunities for promotion, unfair distribution of tasks, employment below one’s own qualification level, etc.) [15]. Studies of interpersonal discrimination, defined by discriminatory behavior by one person towards another [16], have hardly been conducted, especially in German-speaking countries. In a study from the UK, more than 66% of Black nurses and more than half of nurses of Asian origin reported that they were targets of discrimination by patients and patients’ family members [17]. Other studies also identified patients and their family members as source of discrimination [18–20]. Although less investigated, studies show that caregivers from other marginalized groups also experience discrimination in the workplace, suggesting that, for example, lesbian, gay, bisexual and transgender (LGBT) nurses [21], male nurses [22], older nurses, and nurses with a physical disability or limitation [23], may encounter prejudice, (role) stereotyping, belittling, and rejection.

The Everyday Discrimination Scale (EDS) is a widely used instrument for the assessment of perceived discrimination in health-related and social psychological research [24] and the instrument of choice for assessing racial discrimination aimed to capture small unfair acts that occur routinely and are likely to be potentially experienced by everyone. Consisting of nine 5-point Likert-scaled items ranging from 0 (never) to 4 (very often), the EDS asks respondents to indicate the frequency with which they experience various forms of discrimination, such as being treated with less respect or being threatened or harassed. If participants state to have experienced one or more situations, they are also asked to indicate the main reason to which they attribute these experiences. They can choose from a list of several characteristics. This makes the EDS suitable not only for assessing racial discrimination but also for all individuals who can potentially be victims of discrimination based on different factors [24–26].

The EDS has been shown to have a good internal consistency, with Cronbach’s alpha coefficients usually ranging above 0.80 [23] [27–29]. A few studies have also examined the factor structure of the EDS by means of confirmatory factor analysis (CFA) or by using an item response theory (IRT) approach. The results of most of these studies suggest a single-factor model of everyday discrimination, showing a good model fit and overall high factor loadings for all nine items (e.g., [29–31]). Some studies have also found evidence for a two-factor measurement model. For example, Freitas et al. suggested a measurement model for the Portuguese adaptation of the EDS, in which items 1, 2, 7 and 8 load on a “unfair treatment” factor and items 3, 4, 5 and 6 are purported to load on a “personal rejection” factor [32]. To the best of the authors’ knowledge, no adaption of the EDS to health care staff in general and nursing care staff in particular exists. The aim of the present study was to translate and adapt the EDS to nursing staff in Germany and to examine its reliability and factorial validity as well as its measurement equivalence between men and women and different age groups.

## Methods

### Study design and data collection

The study is based on a cross-sectional online survey conducted among health care staff of two hospitals and two inpatient care facilities in Germany. Data collection took place from July to October 2022. All individuals currently working in the field of nursing (health care and nursing staff, pediatric nurses, geriatric nurses, nursing assistants/nursing aides) in one of the aforementioned facilities who were at least 18 years old and provided direct patient care were eligible to participate. Based on common guidelines for confirmatory factor analyses [33] and also guided by a sample size calculation [34], we aimed for a conservative sample size of 300 in order to examine the model fit with a power of at least 80% at a significance level of alpha = 5%. Study participants were recruited through the nursing directors of the respective facilities. They distributed the study information sheet to staff, which provided details on the scope and aims of the study and included the link/QR code for online participation. In addition, further individual participants were recruited via snowballing, by sending e-mails and an information sheet over several mailing lists. The online survey was implemented via the online tool ‘Unipark’ provided by the company Tivian (formerly: Questback).

### Measures

For the purpose of this study, the EDS was translated from English into German using a forward-backward translation approach by two independent researchers with both proficiency in English and German following standard guidelines for the adaptation of survey inventories [35]. Given that the third item of the original EDS refers to experienced service quality (“You receive poorer service than other people at restaurants or stores”), we decided to omit it from the adapted version considering that health care workers provide rather than receive services. The translated items are listed in Table 1.

**Table 1:** Items of the German-language Everyday Discrimination Scale, adapted to nursing staff

Item number	Item (German)	Item (English)
1.	Sie werden von Patient:innen/Bewohner:innen weniger höflich behandelt als Ihre Kolleg:innen	You are treated with less courtesy by patients/residents than your colleagues.
2.	Sie werden von Patient:innen/Bewohner:innen mit weniger Respekt behandelt als Ihre Kolleg:innen	You are treated with less respect by patients/residents than your colleagues.
3.	Patient:innen/Bewohner:innen verhalten sich so, als würden sie denken, Sie seien nicht intelligent	Patients/residents act as if they think you are not smart.
4.	Patient:innen/Bewohner:innen verhalten sich so, als hätten sie Angst vor Ihnen	Patients/residents act as if they are afraid of you.
5.	Patient:innen/Bewohner:innen verhalten sich so, als würden sie Sie für unehrlich halten	Patients/residents act as if they think you are dishonest.
6.	Patient:innen/Bewohner:innen tun so, als wären sie etwas Besseres als Sie	Patients/residents act as if they’re better than you are.
7.	Sie werden von Patient:innen/Bewohner:innen beschimpft oder beleidigt	Patients/residents call you names or insult you.
8.	Sie werden von Patient:innen/Bewohner:innen bedroht oder belästigt	You are threatened or harassed by patients/residents.

Aside from the adapted EDS, the research instrument used for the validation study included questions on *sex*, *age*, *marital status* (single, married/partnership, divorced), *country of birth* (Germany, other) and the *first language spoken* (German, other), *type of facility* respondents work in (hospital, nursing home/other), *occupational position* (senior /deputy senior position), *type of occupation* (nursing, pediatric nursing, elderly care, other) and *type of employment* (full-time, part-time).

### Statistical analysis

For purposes of sample description, chi-square tests were calculated to test for differences between men and women. Means, standard deviations (SD), skewness and kurtosis were calculated for each of the 8 items of the adapted EDS. In addition, inter-item correlations and the item-total correlation for each item are reported. To account for the moderate degree of skewness and kurtosis (particularly in items 4 and 5), robust maximum likelihood (MLR) confirmatory factor analysis (CFA) was used to examine the factor structure of the adapted EDS [33]. We tested a one-dimensional measurement model as most frequently proposed for the EDS in previous studies [29;30]. In addition, we tested a two-factor model as proposed by Freitas et al. for the Portuguese adaptation of the EDS, in which items 1, 2, 7 and 8 are purported to load on an “unfair treatment” factor and items 3, 4, 5 and 6 are purported to load on a “personal rejection” factor [32]. In accordance with established guidelines, multiple indicators, multiple causes (MIMIC) models were used to assess differential item functioning (DIF) related to age and sex [33].

The comparative fit index (CFI), the Tucker-Lewis index (TLI) and the standardized root mean square residual (SRMR) were used to assess the model fit, with TLI and CFI values greater than 0.90 and SRMR values less than 0.08 indicating adequate fit. Furthermore, the root mean square error of approximation (RMSEA) was computed, with values less than 0.08 considered to indicate an acceptable model fit [36]. Items with completely standardized factor loadings below 0.40 were considered for deletion. Modification indices were used to identify sources of model ill-fit. Only those improvements to the model were implemented which were considered theoretically sound [33].

As measures of internal consistency, a composite reliability estimate is reported alongside Cronbach’s alpha with estimates larger than 0.70 regarded as a threshold for acceptable reliability in the latent factor [33]. Cronbach’s alpha is only reported to facilitate comparisons with other studies in the field and should be interpreted cautiously considering its limitations [37].

Descriptive analyses were performed using Stata 15 [38]. The R package lavaan 0.6-3 [39] was used to conduct the CFA and MIMIC analysis.

## Results

Data on 302 individuals were available, of whom 65 (21.5%) were men and 237 (78.5%) were women. Almost 60% of the sample was 40 years of age or younger. Nearly 90% were born in Germany and reported German as their first language, with the latter slightly differing between men and women. A total of 82.8% worked in a hospital, and 17.2% reported working in a nursing home or other setting. The majority were general or pediatric nurses (61.3% and 11.6%, respectively), and 9.3% specialized in elderly care. Most reported working full-time and in non-senior positions (70.9% and 67.5%, respectively) (Table 2). The proportion of full-time employees was significantly higher for males than females (86.2% vs. 66.7%).

**Table 2:** Description of the study sample by sex (nursing staff in Germany; n=302)

Variable	Sex		p-value
	Male (n = 65)	Female (n = 237)	
**Age (in years)**			**0.28**
18–20	2 (3.1%)	5 (2.1%)	
21–30	15 (23.1%)	63 (26.6%)	
31–40	27 (41.5%)	67 (28.3%)	
41–50	8 (12.3%)	45 (19.0%)	
51–60	13 (20.0%)	51 (21.5%)	
≥61	0 (0.0%)	6 (2.5%)	
**Marital status**			**0.41**
Single	37 (56.9%)	117 (49.4%)	
Married/partnership	22 (33.8%)	102 (43.0%)	
divorced	6 (9.2%)	18 (7.6%)	
**Country of birth**			**0.09**
Germany	61 (93.8%)	204 (86.1%)	
Other	4 (6.2%)	33 (13.9%)	
**First language spoken**			**0.03**
German	62 (95.4%)	202 (85.2%)	
Other	3 (4.6%)	35 (14.8%)	
**Type of occupation**			**0.09**
Nursing	46 (70.8%)	139 (58.6%)	
Pediatric Nursing	2 (3.1%)	33 (13.9%)	
Elderly care	6 (9.2%)	22 (9.3%)	
Other	11 (16.9%)	43 (18.1%)	
**Occupational position**			**0.38**
Senior/deputy senior position	24 (36.9%)	74 (31.2%)	
Non-senior position	41 (63.1%)	163 (68.8%)	
**Type of facility**			**0.30**
Hospital	51 (78.5%)	199 (84.0%)	
Nursing home / other	14 (21.5%)	38 (16.0%)	
**Type of employment**			**< 0.01**
Full-time	56 (86.2%)	158 (66.7%)	
Part-time	9 (13.8%)	79 (33.3%)	

*Note:* Because of rounding not all percentages add up to 100%. * p-value from chi-square test

Basic statistics for the 8 items of the adapted EDS are reported in Table 3. Some of the items, particularly items 4 and 5, experienced a moderate degree of skewness and kurtosis. Inter-item correlations and the item-total correlation for each item are reported in Table 4.

**Table 3:** Descriptive statistics for the 8 items of the German-language Everyday Discrimination Scale (nursing staff in Germany, n = 302)

Item	Mean	SD	Skewness	Kurtosis
1	2.33	1.41	0.97	3.24
2	2.19	1.21	0.98	3.64
3	2.24	1.29	1.12	3.85
4	1.61	0.96	1.96	7.46
5	1.75	1.05	1.55	5.21
6	2.87	1.42	0.50	2.63
7	2.81	1.35	0.57	2.91
8	2.28	1.20	0.96	3.65

*Notes*: See Table 1 for item content. SD: standard deviation

**Table 4:** Inter-item and item-total correlations of the 8 items of the German-language Everyday Discrimination Scale (nursing staff in Germany, n = 302)

	Inter-item correlation (Pearson correlation coefficients)								Item-total correlation
	Item 1	Item 2	Item 3	Item 4	Item 5	Item 6	Item 7	Item 8	
Item 1	1								0.65
Item 2	0.69	1							0.70
Item 3	0.59	0.58	1						0.69
Item 4	0.36	0.35	0.33	1					0.43
Item 5	0.38	0.40	0.53	0.52	1				0.61
Item 6	0.52	0.56	0.63	0.24	0.52	1			0.71
Item 7	0.44	0.53	0.51	0.30	0.46	0.63	1		0.69
Item 8	0.34	0.44	0.33	0.19	0.34	0.53	0.70	1	0.55

**Fig. 1:**
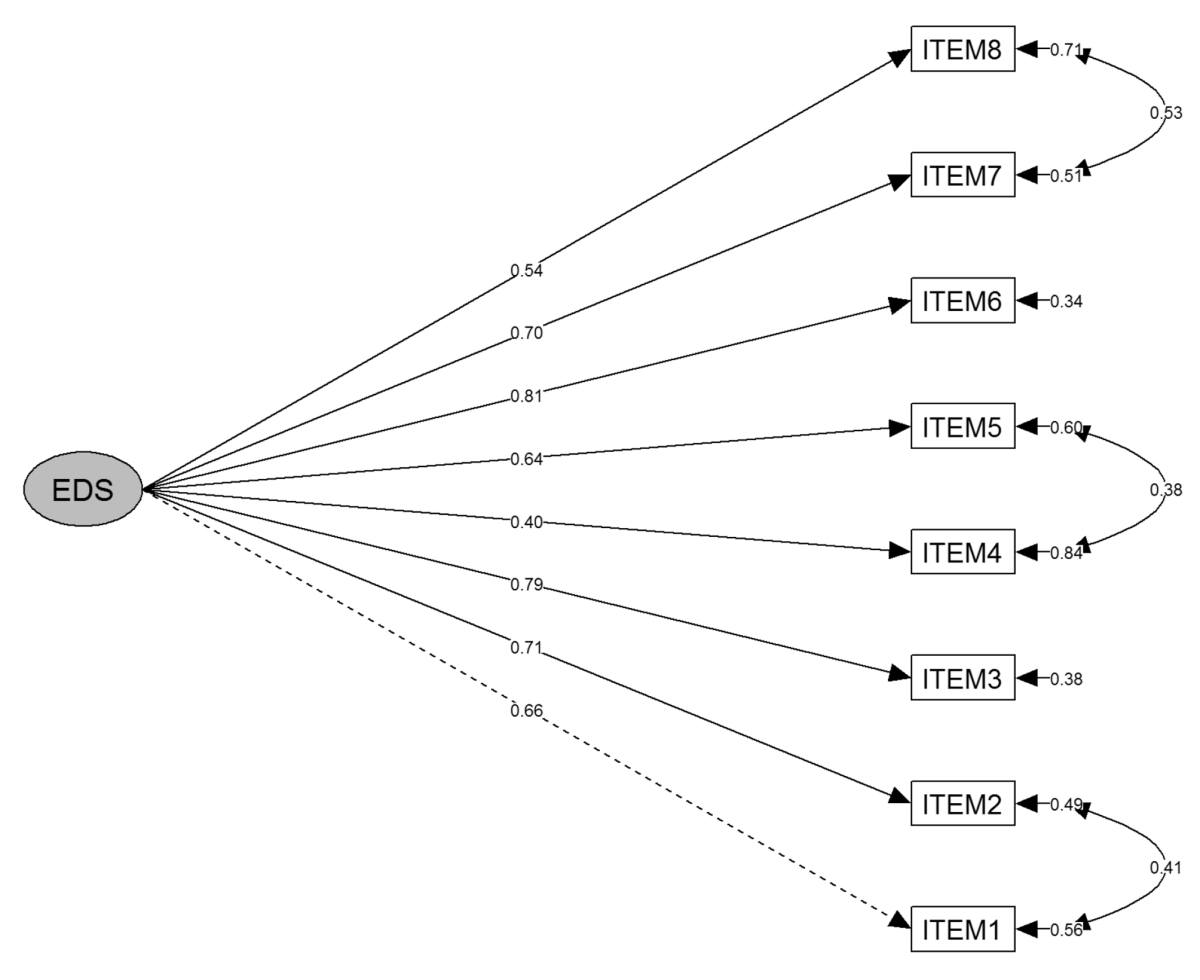
Factor structure of the German-language Everyday Discrimination Scale (nursing staff in Germany; completely standardized factor loadings, residual variances and covariances are represented by the numbers on the straight and curved arrows, respectively; n = 302; All factor loadings/covariances were significant at p < 0.001)

The one-factor, 8-item baseline model of the adapted EDS showed a poor fit (RMSEA = 0.149; CFI = 0.812; TLI = 0.737; SRMR = 0.072). After the addition of three error covariances between items 1 (“You are treated with less courtesy by patients/residents than your colleagues”) and 2 (“You are treated with less respect by patients/residents than your colleagues.”), items 4 (“Patients/residents act as if they are afraid of you.”) and 5 (“Patients/residents act as if they think you are dishonest.”) and items 7 (“Patients/residents call you names or insult you.”) and 8 (“You are threatened or harassed by patients/residents.”) (Fig. 1) the model fit improved considerably (RMSEA = 0.066; CFI = 0.969; TLI = 0.949; SRMR = 0.036). Item 4 had a low completely standardized factor loading (λ) of 0.40; given that it was not below 0.40, it was retained in the measurement model following common guidelines [33]. All other factor loadings were ≥0.5. All factor loadings were significant at p < 0.001. The two-factor alternative measurement model as reported in the Portuguese validation study [32] showed a poor model fit (RMSEA = 0.152; CFI = 0.815; TLI = 0.727; SRMR = 0.070).

Cronbach’s alpha for the one-factor model was 0.87, with the respective composite reliability estimate being 0.86.

Item 4 showed differential item functioning (DIF) related to sex and age, as evidenced by significant direct effects of sex and age on the item while holding the latent factor constant. DIF related to age could also be observed for item 6. The effects (β=-0.192, β = 0.147 and β=-0.156, respectively), were moderate in size and did not bias the comparison between men and women or between younger and older employees. Irrespective of adjusting for DIF related to sex and age, no significant difference in perceived discrimination was observed between male and female nursing staff, while older individuals had a lower perceived discrimination score than younger individuals (β=-0.213, p < 0.001).

## Discussion

The EDS is a widely used survey inventory in health and social psychology research to examine individuals’ perceptions of discrimination [24], with a particular focus on capturing everyday instances of unfair treatment based on a variety of diversity characteristics. The aim of the present study was to translate the EDS to German and to adapt and validate it for the assessment of discrimination experiences of nursing staff.

Our analysis found that the one-factor model as the most commonly used measurement model of the EDS only had a moderate fit when applied to nursing staff in Germany. However, the fit improved significantly when we added three error covariances in a reparameterized model. Previous research on the factor structure of the EDS in other population groups also found that adding error covariances can improve the model’s fit. In a study on everyday discrimination and substance use among adolescents in Northern Chile, Caqueo-Urizar et al. also showed that parameterizing error covariances between items 1 and 2 and items 7 and 8 can drastically improve the model fit of the EDS [40]. Similarly, Freitas et al., although identifying a two-dimensional structure, had to include error covariances between items 1 and 2 and items 7 and 8 to improve the model fit of the scale. Based on a large sample of African Americans and employing an IRT framework, Stucky et al. also identified local dependency in items 4 and 5 and items 7 and 8 as well as an item context effect for items 1 to 3 [31]. The need to add error covariance is probably the result of the items being presented in a series and being conceptually similar, which is particularly the case for items 1 and 2 on “courtesy” and “respect”. As pointed out by Stucky et al., items 4 (“afraid”) and 5 (“dishonest”) are linked to perceptions of distrust towards the respondent. Similarly, items 7 (“insult”) and 8 (“harass”) are related to visible/direct aggression. Given that the purpose of the EDS is especially to assess indirect forms of discrimination that occur on a daily basis, Stucky et al. propose to exclude such items from the EDS, something that should be further evaluated in future studies. Furthermore, it needs to be considered that particularly item 7 and 8 of the EDS each simultaneously refer to two different phenomena (item 7: name calling and insults; item 8: threats and harassment) and it may be unclear for respondents what to focus on when answering the questionnaire.

The need to add error covariances to improve the fit of the model as identified in this and previous investigations could also indicate some underlying conceptual problems in the factor structure of the EDS, which should be investigated in future psychometric studies. Amongst others, these studies should also examine whether the response categories of the EDS are equidistant. While we based the inclusion of error covariances on theoretical considerations and although they resemble modifications similar to those used in previous research, these post hoc modifications must be treated as exploratory changes to the measurement model which must be cross-validated in other populations and languages. With the three error covariances implemented, the translated EDS can be considered a valid instrument for the assessment of discrimination experiences among nursing staff. Our analysis, however, showed that some items are prone to sex-related DIF. This is consistent with findings from other studies [31]. Although the magnitude of DIF in our study was small, it is necessary to account for this bias in order to accurately compare discrimination experiences between male and female health care workers. Latent variable modelling is a useful approach for this purpose and also allows for the incorporation of adjustments resulting from error covariances [33].

To the best of the authors’ knowledge, this is the first study to translate the EDS to German and the first to adapt and validate it for nursing staff. Some limitations of the study need to be considered. Sampling took place in only four facilities and the initial sample size was comparably small. On the one hand, this can be attributed to the sensitive nature of the survey topic. On the other hand, the still present restrictions related to the COVID-19 pandemic and study participants being burdened by many other ongoing, particularly COVID-19-related, surveys as well as a high workload in the health care setting may have reduced participants’ capacities to take part in the study. This assumption was also confirmed by some facilities which approached us after the survey. In order to increase the sample size, we recruited additional participants by means of a snowball sampling approach. Given that this approach was similar to the first approach where potential participants have been recruited through information leaflets distributed by the nursing directors of the respective facilities, we do not consider this a major source of bias. Nevertheless, both approaches to data collection, including the small number of facilities in which sampling took place, have to be regarded convenience sampling with its well-known limitations [41]. In our study we focused on nursing staff. Future investigations need to examine whether our findings can be generalized to other groups of healthcare workers, such as physicians.

## Conclusion

Discrimination in health care settings can have detrimental effects on the mental and physical health of health care professionals, including nursing staff. Despite the growing recognition of the negative impact of such experiences, the availability of validated tools for assessing discrimination among health care professionals remains limited. The present study shows that the adapted EDS can be considered a valid instrument for the assessment of discrimination experiences among nursing staff, if some modifications to the original measurement model are implemented. It has the potential to provide robust data on the various forms of discrimination and violence to which health care professionals in general and nursing staff in particular are exposed. The insights gained are relevant for both research and practice. They allow theoretical concepts from discrimination research to be transferred to new settings and, if necessary, to be adapted and expanded. Furthermore, the identification of experiences of discrimination among health professionals can contribute to the development of adequate organizational strategies as well as health-related interventions that avoid or reduce the negative consequences of discrimination and promote the mental well-being of those affected. The development of such interventions can benefit from the availability of validated assessment tools like the EDS. Future studies, among others, need to examine the convergent and discriminant validity of the adapted EDS and should also investigate its performance in other language and health care contexts.

## Data Availability

This study utilizes confidential patient data protected by the German Data Privacy Act. In order to ensure patient confidentiality and privacy, the data used in this study is only available in anonymous and aggregated form. Researchers who are interested in accessing the anonymized data must obtain approval from the Local Psychological Ethics Committee at the Center for Psychosocial Medicine (LPEK), Hamburg, Germany and should contact the corresponding author for that purpose.

## Abbreviations

DIF: Differential item functioning
CFA: Confirmatory factor analysis
CFI: Comparative fit index
EDS: Everyday Discrimination Scale
IRT: Item response theory
MIMIC: Multiple indicators multiple causes
MLR: Robust maximum likelihood
RMSEA: Root Mean Square Error of Approximation
TLI: Tucker-Lewis-Index
SRMR: Standardized root mean square residual
